# Exploration of dilated cardiomyopathy for biomarkers and immune microenvironment: evidence from RNA-seq

**DOI:** 10.1186/s12872-022-02759-7

**Published:** 2022-07-18

**Authors:** Chenggang Fang, Zhan Lv, Zhimin Yu, Kexin Wang, Chengkai Xu, Yixuan Li, Yanggan Wang

**Affiliations:** 1 Department of Internal Medicine, Zhongnan Hospital of Wuhan University, Wuhan, China; 2 Medical Research Institute of Wuhan University, Wuhan, China

**Keywords:** Dilated cardiomyopathy, DCM, Hub genes, Biomarker, Immune microenvironment

## Abstract

**Background:**

The pathogenic mechanism of dilated cardiomyopathy (DCM) remains to be defined. This study aimed to identify hub genes and immune cells that could serve as potential therapeutic targets for DCM.

**Methods:**

We downloaded four datasets from the Gene Expression Omnibus (GEO) database: GSE141910, GSE3585, GSE42955 and GSE79962. Weighted gene coexpression network analysis (WGCNA) and differential expression analysis were performed to identify gene panels related to DCM. Meanwhile, the CIBERSORT algorithm was used to estimate the immune cells in DCM tissues. Multiple machine learning approaches were used to screen the hub genes and immune cells. Finally, the diagnostic value of the hub genes was assessed by receiver operating characteristic (ROC) analysis. An experimental mouse model of dilated cardiomyopathy was used to validate the bioinformatics results.

**Results:**

FRZB and EXT1 were identified as hub biomarkers, and the ROC curves suggested an excellent diagnostic ability of the above genes for DCM. In addition, naive B cells were upregulated in DCM tissues, while eosinophils, M2 macrophages, and memory CD4 T cells were downregulated in DCM tissues. The increase in two hub genes and naive B cells was validated in animal experiments.

**Conclusion:**

These results indicated that FRZB and EXT1 could be used as promising biomarkers, and eosinophils, M2 macrophages, resting memory CD4 T cells and naive B cells may also affect the occurrence of DCM.

**Supplementary Information:**

The online version contains supplementary material available at 10.1186/s12872-022-02759-7.

## Introduction

Dilated cardiomyopathy (DCM) is defined as left ventricular (LV) dilatation and left ventricular systolic dysfunction in the absence of abnormal loading conditions (hypertension, valve disease) or coronary artery disease sufficient to cause global systolic impairment [1]. DCM is one of the most common causes of heart failure, and its prevalence ranges from 1:250 to 1:2500 in the general population [2, 3]. If patients are not promptly treated, the 1-year survival rate is 70–75% and the 5-year survival rate is as low as 50% [4]. Causes of DCM can be classified into two categories, genetic and nongenetic, but overlap exists within the two categories. Under the influence of various physicochemical factors, the most common reactive changes include inflammation (viral myocarditis or autoimmune disease), nutritive-toxic influences (alcohol, drugs, chemotoxins), and metabolic disorders. These changes finally lead to remodelling of the myocardium [5]. However, the molecular mechanism underlying remodelling is a complex network of cellular signalling pathways and is not fully understood. Genetic testing for cardiovascular disease has become more common in recent years. The detection rate of hypertrophic cardiomyopathy is approximately 60–70%, while that of dilated cardiomyopathy is much lower than 60%. It has been shown that among the genes encoding structural proteins, mutations are more frequent in genes encoding structures such as the cytoskeleton, cardiomyocyte sarcomere and nuclear membrane proteins [2, 6, 7]. Therefore, it is essential to identify novel biomarkers significantly correlated with the DCM diagnosis to improve the effectiveness of therapeutic approaches.

The combination of myocardial inflammation (myocarditis) and dysfunction is termed inflammatory cardiomyopathy. In patients with recent-onset DCM, the identification of myocarditis has important clinical implications due to the high potential for LV recovery. Circulating cardiac autoantibodies are more common in patients with dilated cardiomyopathy and myocarditis than in patients with noninflammatory heart disease.

Furthermore, in healthy relatives of patients with dilated cardiomyopathy, serum anti-heart autoantibodies are independent predictors of disease progression [8]. Both innate and adaptive immunological aspects may play a role in affecting outcomes in laboratory animals and patients with viral myocarditis [9]. The failing myocardium can provide signals to assist in immune cell infiltration via upregulation or secretion of various cytokines, such as P-selectin, e-selectin, intracellular cell adhesion molecule-1, and vascular cell-adhesion molecule-1, which allows transendothelial migration of a range of immune cells into the myocardium, including B cells, T cells, natural killer cells, monocytes, and platelets [10, 11]. If we can determine the most relevant type of immune cells, a novel treatment may be possible by interfering with a particular type of immune cell.

In this research, we examined 4 GEO datasets and found 3743 significant differentially expressed genes (DEGs) between normal and DCM samples. A weighted gene coexpression network analysis (WGCNA) was performed to evaluate the key module correlated with DCM. LASSO tenfold cross-validation was used to further knockout redundant genes, and 38 potential genes were finally screened out. SVM machine learning, random forest tree and logistic analyses were used to conduct in-depth screening, and FRZB and EXT1 were identified as hub biomarkers. In the screening set, ROC and difference analyses were performed on the above two genes. The results showed that the two genes had good predictive performance in the screening set. Random forest tree analysis and the Wilcoxon rank-sum test were performed to identify core immune cells that may affect the occurrence of DCM, and four types of immune cells were ultimately selected.

## Materials and methods

### Datasets and data preprocessing

The GSE141910 dataset (FPKM format) based on the GPL16791 platform (166 healthy myocardial tissue samples and 166 DCM samples), which is the original RNA-seq dataset downloaded from the GEO database [12], was used as a screening set.

In addition, the GSE3585 dataset (GPL96, 5 normal samples and 7 DCM samples), GSE42955 dataset (GPL6244, 5 normal samples and 12 DCM samples), and GSE79962 dataset (GPL6244, 11 normal samples and 9 DCM samples) were collectively defined as an external verification set, and the sva package[13] was used to perform background correction, normalization and expression calculation on the original data (Addtional file 1: Figure S1).

### Screening of hub biomarkers

The limma package [14] was used to identify differentially expressed genes (DEGs) in the screening set, and |log2-fold change FC|> 0.5 and adj. *P* value < 0.05 were selected as cut-off criteria. In the WGCNA [15], all DEGs were used as input, and topological calculations were performed with a soft threshold value of 1 to 20. According to the optimal soft threshold value, the relation matrix wasis converted into an adjacent matrix and then converted into a topological overlap matrix (TOM). Average-linkage hierarchical clustering was performed, related modules were classified according to the TOM, the number of genes in each module was not less than 50, and similar modules were merged. Then, the Pearson method was used to calculate the correlation between the merged module and DCM. Among the core modules screened by the WGCNA, LASSO tenfold cross-validation was performed to knockout redundant genes (glmnet package) [16]. Subsequently, the support vector machine-recursive feature elimination (SVM-REF) [17, 18] method (taking the lowest point feature of the RSM), the random forest tree method (taking the genes with the top 10 weights), and the single-factor logistic regression method (taking the top 3 OR values) were adopted to screen the nonredundant genes. Ultimately, the genes identified with the above methods were overlapped to identify the final core gene.

### Enrichment analysis

GO enrichment analysis is a commonly used bioinformatics method for searching comprehensive information of large-scale genetic data, including BP, CC, and MF. In addition, KEGG pathway enrichment analysis is widely used to understand biological mechanisms and functions. Furthermore, DO enrichment analysis can explore the diseases in which the relevant genes are mainly involved. The GOplot [19] package was used to visualize the GO, KEGG pathways, and DO analysis. Finally, the clusterprofile package [20] and GSVA package [21] were used to further explore the important signalling pathways related to core genes. The h.all.v7.4.symbols gene set was downloaded from MSigDB [22], and GSEA was performed on the gene set and gene expression matrix to explore the regulatory pathways that may be involved.

### Construction of regulatory network

First, the mirDIP database [23] was used to predict potential miRNAs targeting hub genes and identify miR regulatory networks. The TF-core gene interaction pair with P < 0.05 in the TRRUST database [24] was selected to establish an upstream regulatory network. Then, we searched the Comparative Toxicogenomics database [25] for compounds that may be potentially related to core genes. Finally, the Network Analyst database [26] was used to visualize the core gene regulation network.

### CIBERSORT algorithm

The CIBERSORT algorithm [27] calculates the proportion of different immune cell types based on the expression levels of immune cell-related genes. The output results of 22 infiltrated immune cells were integrated to generate a matrix of immune cell components for analysis (CIBERSORT package).

### Screening of hub immune cells

The Wilcoxon test was used to investigate the differences in the content of immune cells in different tissues. Meanwhile, the randomForest package was used to construct a random forest tree of 22 kinds of immune cells, determine the points with the smallest error, sort the immune cells according to their importance, and then select the immune cells with an importance score greater than 10. Ultimately, the immune cells identified with the above method were overlapped, and the core immune cells that regulate the occurrence of DCM were screened out.

### Experimental mouse model of dilated cardiomyopathy

Male C57/BL6 mice aged 6–8 weeks were used in accordance with the animal protocol specifically approved for this study by the Wuhan University Animal Care and Use Committee.

The mice in the DCM group were intraperitoneally administered Dox solution at a dose of 5 mg/kg using a 1 mL sterilized syringe once a week. The control group was treated with the same amount of saline solution according to the same method.

The body weights of the two groups of mice were measured to adjust the injection dose for a total of 4 weeks, with a cumulative dose of 20 mg/kg.

### Quantitative RT–PCR

RNA was isolated using the RNA prep fast pure tissue kit (TsingKe Biotechnology). cDNA was synthesized using the RevertAid First Strand cDNA Synthesis Kit (Thermo Fisher Scientific). Quantitative RT–PCR was performed by Bio-Rad CFX96 Touch using a SYBR green (Roche)-based assay. GAPDH was employed as an internal control. Quantitative RT–PCR was performed with the specific primers shown in Table 1.

**Table 1:** Primer sequences for the target gene for RT–qPCR

Gene	Primer sequence
*EXT1*	
Forward	TGCCACTTTCTGTCTGGTTCCT
Reverse	AATCACTTCGGAGAATGGCAAC
*Frzb*	
Forward	TAAACATTCCAAGGGACACCGT
Reverse	AGAGCCTTCTACCAAGAGTAACCTG
*GAPDH*	
Forward	CCTCGTCCCGTAGACAAAATG
Reverse	TGAGGTCAATGAAGGGGTCGT

### Echocardiography

Echocardiography was performed to evaluate the heart condition of mice with a Vevo 2100 imaging system (VisualSonics). The data were obtained from M-mode with a stable heart rate from 500 to 600 bpm.

### Immunofluorescence

First, the heart sections were washed with PBS, and the primary antibody was diluted in FACS buffer and added into a hydrated chamber (anti-CD19 rabbit pAb [Servicebio]) overnight at 4 °C. Afterwards, sections were washed with PBS and stained with secondary antibody diluted in PBS for 1 h at 4 °C (Cy3 conjugated goat anti-rabbit IgG [Servicebio]). Sections were subsequently washed with PBS and incubated with DAPI solution for 10 min at room temperature. The sections were washed again with PBS, and then spontaneous fluorescence quenching reagent was added and the sections were incubated for 5 min. Finally, the sections were washed in running tap water for 10 min. Immunofluorescence images were obtained under a microscope (Olympus) at 400 times magnification and analysed with Image-Pro Plus 6.0 software.

### Statistical analysis

All statistical analyses were performed with GraphPad 9.0, and statistical significance was considered at *P* < 0.05.

## Results

### Differentially expressed genes

**Fig. 1:**
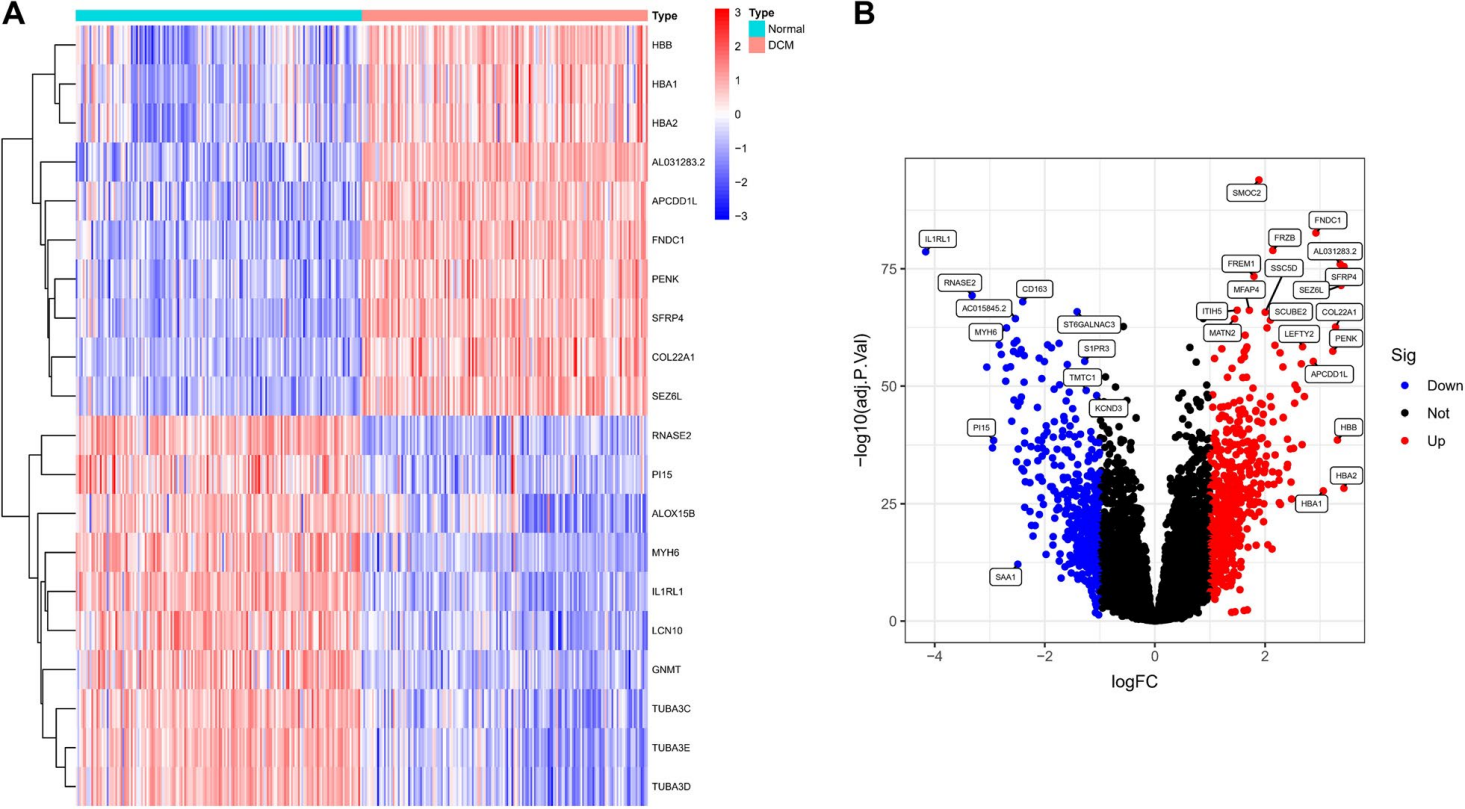
**A** Heatmap of the 20 DEGs between DCM and normal samples (generated by ggplot2); **B** Volcano plot visualizing DEGs between normal and DCM samples

Differentially expressed genes (DEGs) were analysed in the screening set, and 3743 DEGs (Additional file 2) were ultimately identified. The heatmap shows the top 20 DEGs (Fig. 1A). The volcano map shows 1861 upregulated genes and 1882 downregulated genes (Fig. 1B).

#### WGCNA

**Fig. 2:**
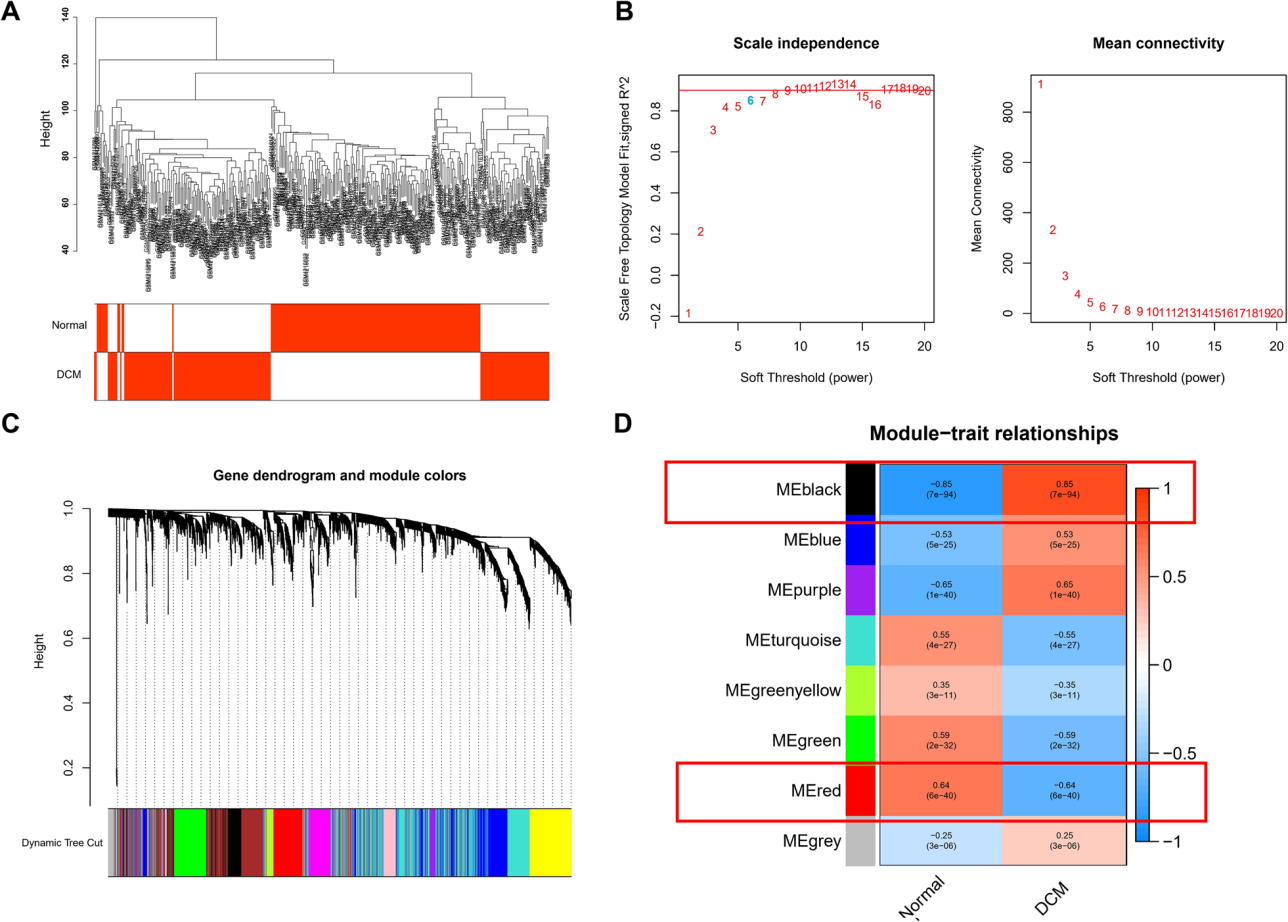
Identification of key modules correlated with clinical traits by WGCNA. **A** Clustering dendrograms of samples; **B** Analysis of the scale-free fit index and the mean connectivity under various soft-thresholding powers; **C** Dendrogram of all DEGs clustered with the topological overlap dissimilarity measure; **D** Heatmap of the correlation between module eigengenes and clinical traits. Each row corresponds to a module eigengene, each column represents a clinical trait, and each cell contains the correlation coefficient and p value (generated by ggplot2)

The clinical information and genes were correlated, and a WGCNA was performed. The clustering situation of each sample was favourable, with no outlier sample (Fig. 2A), and the optimal soft threshold was determined to be 6 (Fig. 2B). The modules were classified according to the soft threshold and the TOM, and the number of genes in each module was not less than 50 (Fig. 2C). Similar gene modules were merged, and 8 modules were finally identified (Fig. 2D). By calculating the correlation between module genes and clinical traits, it was found that the black module containing 1078 genes had the highest positive correlation with the occurrence of DCM (r = 0.85), and the red module containing 265 genes had the highest negative correlation with the occurrence of DCM (r = − 0.64). Using both as core modules, 1343 potential core genes were finally identified, including 834 upregulated genes and 509 downregulated genes (Additional file 3).

### Enrichment analysis

**Fig. 3:**
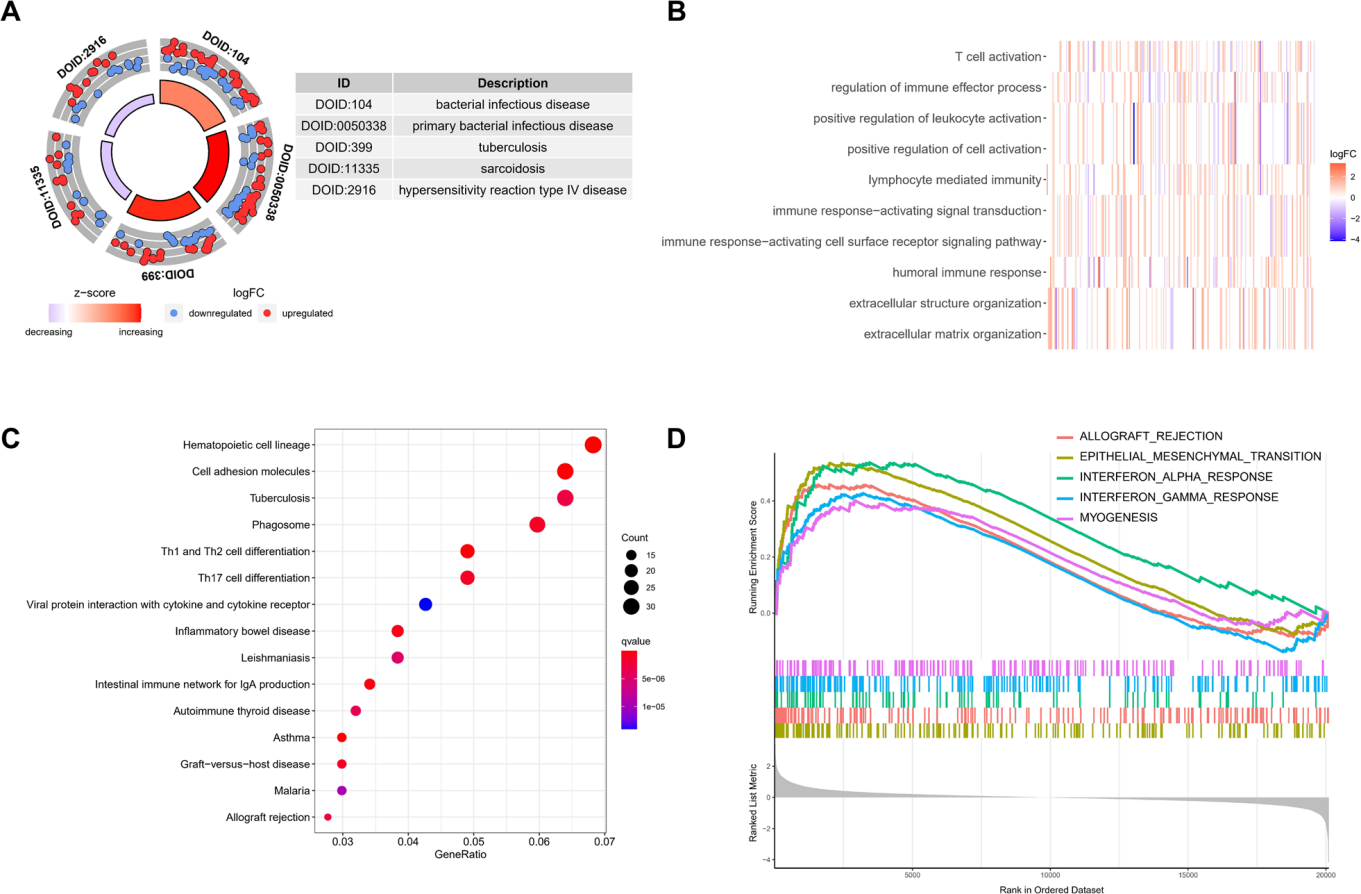
Enrichment analysis of 1343 potential core genes. **A** DO enrichment analysis; **B** GO enrichment analysis; **C** KEGG enrichment analysis; **D** GSEA enrichment analysis

To explore the potential biological mechanism of DCM, enrichment analysis was performed on 1343 potential core genes. DO analysis revealed the types of diseases that may have common pathogenesis, such as bacterial infectious disease, tuberculosis and sarcoidosis (Fig. 3A). Further GO analysis showed that T-cell activation, regulation of immune effector processes, positive regulation of leukocyte activation and other processes were significantly enriched (Fig. 3B). In addition, KEGG also described specific pathways, such as Th1, Th2 and Th17 cell differentiation and viral protein interaction with cytokines and cytokine receptors (Fig. 3C). The above results indicate that immune-related factors may affect the occurrence of DCM. Ultimately, GSEA was performed on the gene set and expression matrix, and the results showed that INTERFERON_ALPHA_RESPONSE, INTERFERON_GAMMA_RESPONSE and other pathways were significantly enriched (Fig. 3D). In summary, the strong chain of evidence indicates the important role of immunity in the pathogenesis of DCM.

### Exploring hub biomarkers

**Fig. 4:**
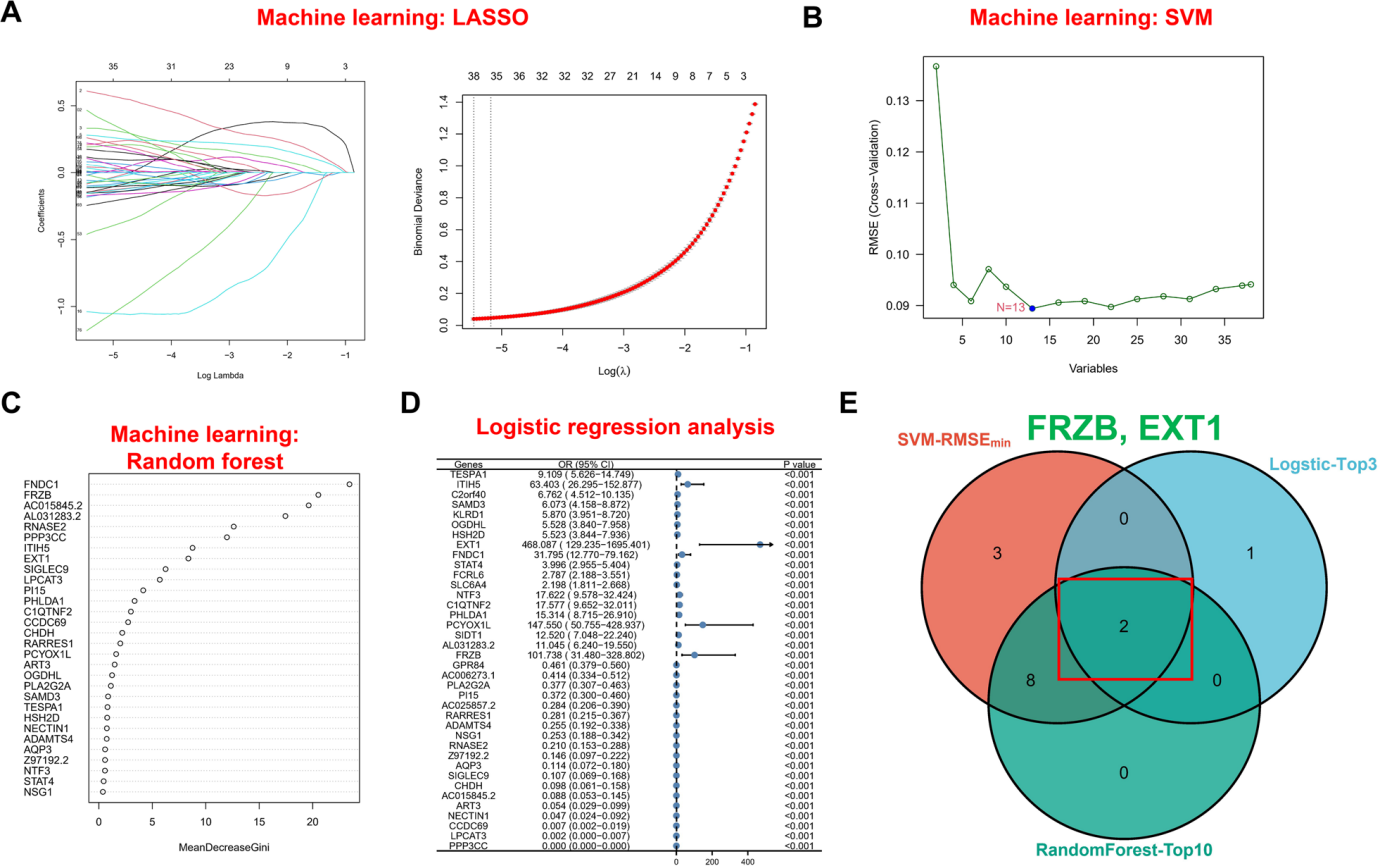
Outcome of multiple machine learning approaches. **A** LASSO regression, **B** SVM, **C** random forest, **D** logistic regression analysis and **E** Venn diagram showing the overlapping genes of three machine learning approaches

First, LASSO tenfold cross-validation was used to further knockout redundant genes, and 38 potential genes were finally screened out (Fig. 4A). Among these 38 genes, the SVM machine learning method was used to conduct in-depth screening. The results showed that when 19 genes were included, the RMSE value was the lowest (Fig. 4B). In addition, the random forest tree method was used to rank the weights of the 38 genes (Fig. 4C). At the same time, the occurrence of DCM was used as the dependent variable, and logistic analysis was performed. The results of the forest plot showed the OR value and confidence interval corresponding to each gene (Fig. 4D). Finally, the genes identified by the above algorithm were overlapped, and FRZB and EXT1 were identified as hub biomarkers (Fig. 4E).

### Validation of hub biomarkers

**Fig. 5:**
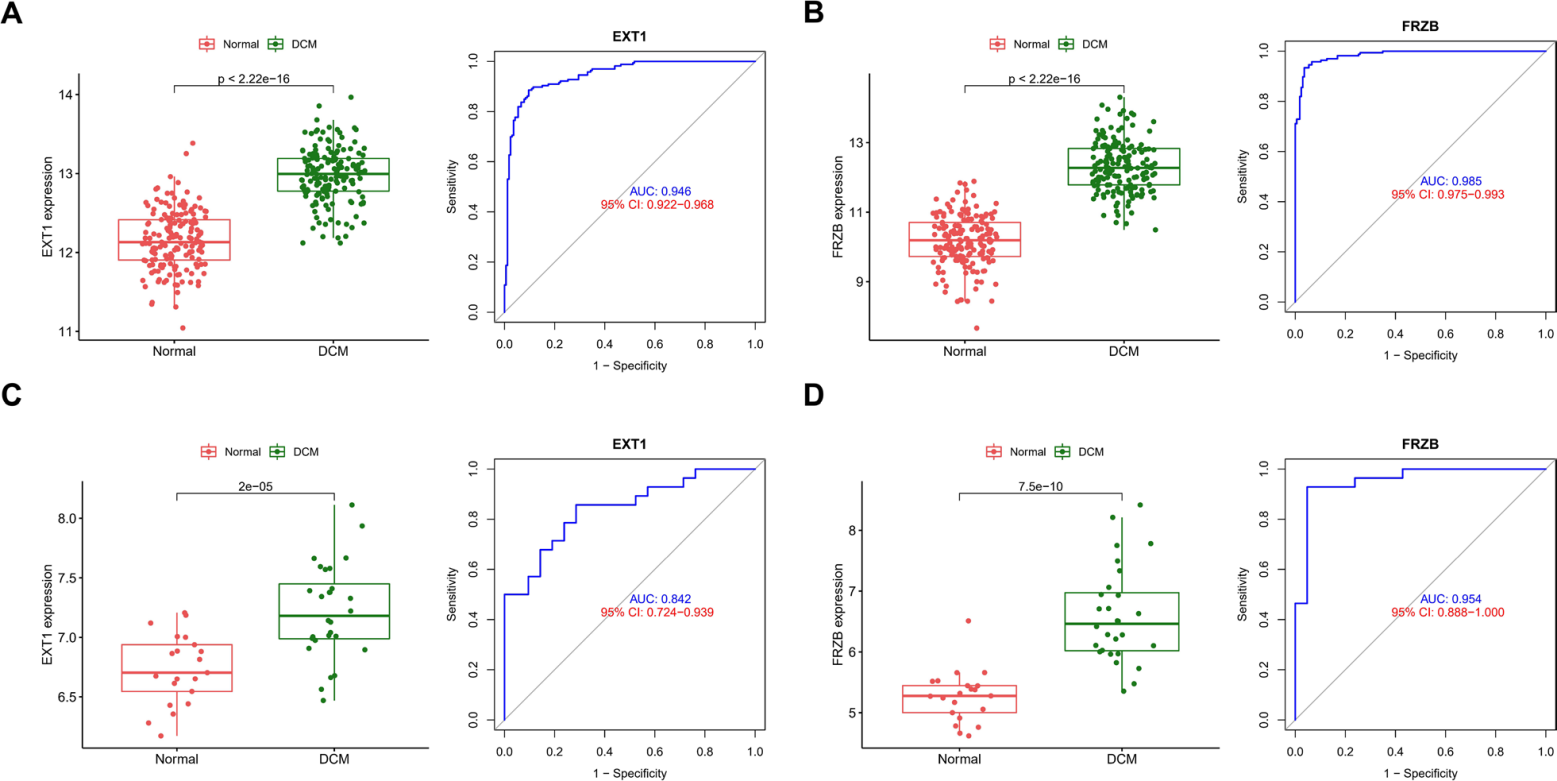
The diagnostic values of hub genes in DCM. ROC curves and AUC statistics to evaluate the diagnostic efficiency of hub genes on the incidence of DCM in the screening set (**A**, **B**) and in the external validation set (**C**, **D**)

In the screening set, ROC and difference analyses were performed on the above two genes. The results showed that the two genes had good predictive performance in the screening set (EXT1 (AUC = 0.946) and FRZB (AUC = 0.985), which were both highly expressed in DCM samples) (Fig. 5A, B). In the external validation set, the expression of core genes was similar to that in the screening set, which were upregulated in DCM tissues with strong diagnostic performance (EXT1, AUC = 0.842; FRZB, AUC = 0.954) (Fig. 5C, D). In addition, the regulatory network of the above two core genes was visualized, a TF-mRNA-miRNA network was constructed, and potential candidate compounds targeting EXT1 and FRZB were predicted to improve the symptoms of DCM patients (Addtional file 1: Figure S2).

### Analysis of differences in the immune microenvironment

**Fig. 6:**
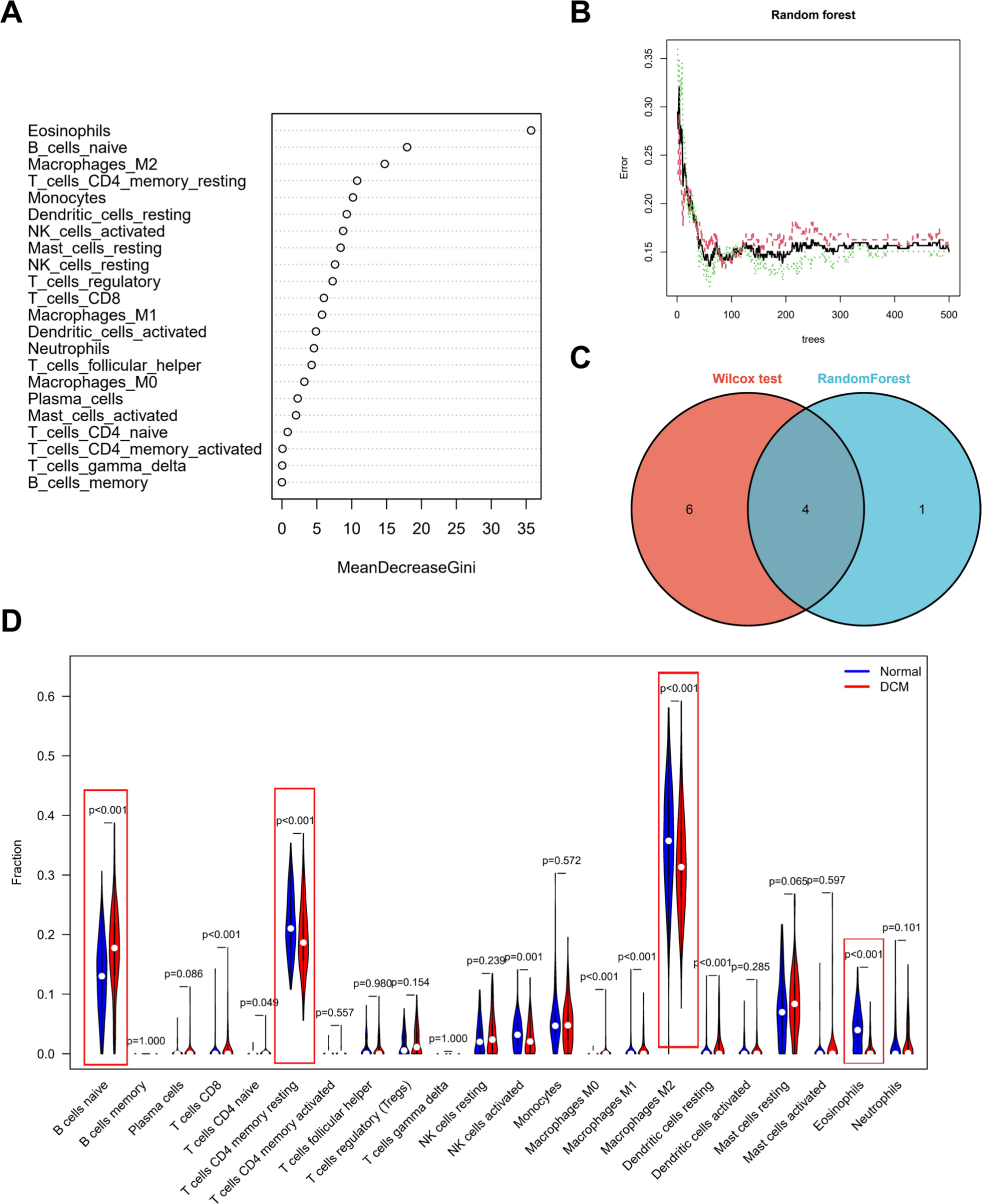
Wilcoxon test (**A**) and random forest tree (**B**) were performed to identify the core immune cells, and a Venn diagram (**C**) was used to show the overlapping immune cells and the fraction of immune cells (**D**)

Considering the important role of immune-related pathways in the occurrence of DCM in gene enrichment analysis (Fig. 2), the CIBERSORT algorithm was used to analyse the content of immune cells in different samples. The histogram shows the overall landscape of immune cell distribution, and the results of the heatmap show in detail the correlation of 22 types of immune cells (Additional file 1: Figure S3). The results of Wilcoxon test analysis showed the difference in the content of immune cells in DCM samples and normal myocardial tissues. To identify the core immune cells that change the immune microenvironment in myocardial tissue, random forest tree analysis was performed on 22 immune cells (Fig. 6A, B). Subsequently, the immune cells identified by the Wilcoxon test and random forest tree were overlapped, and four core immune cells that may affect the occurrence of DCM were finally identified (Fig. 6C): eosinophils, M2 macrophages, resting memory CD4 T cells and naive B cells. Among them, only naive B cells were upregulated in DCM tissues, while eosinophils, M2 macrophages, and memory CD4 T cells were downregulated in DCM tissues (Fig. 6D).

### Correlation analysis of immune cells and hub biomarkers

**Fig. 7:**
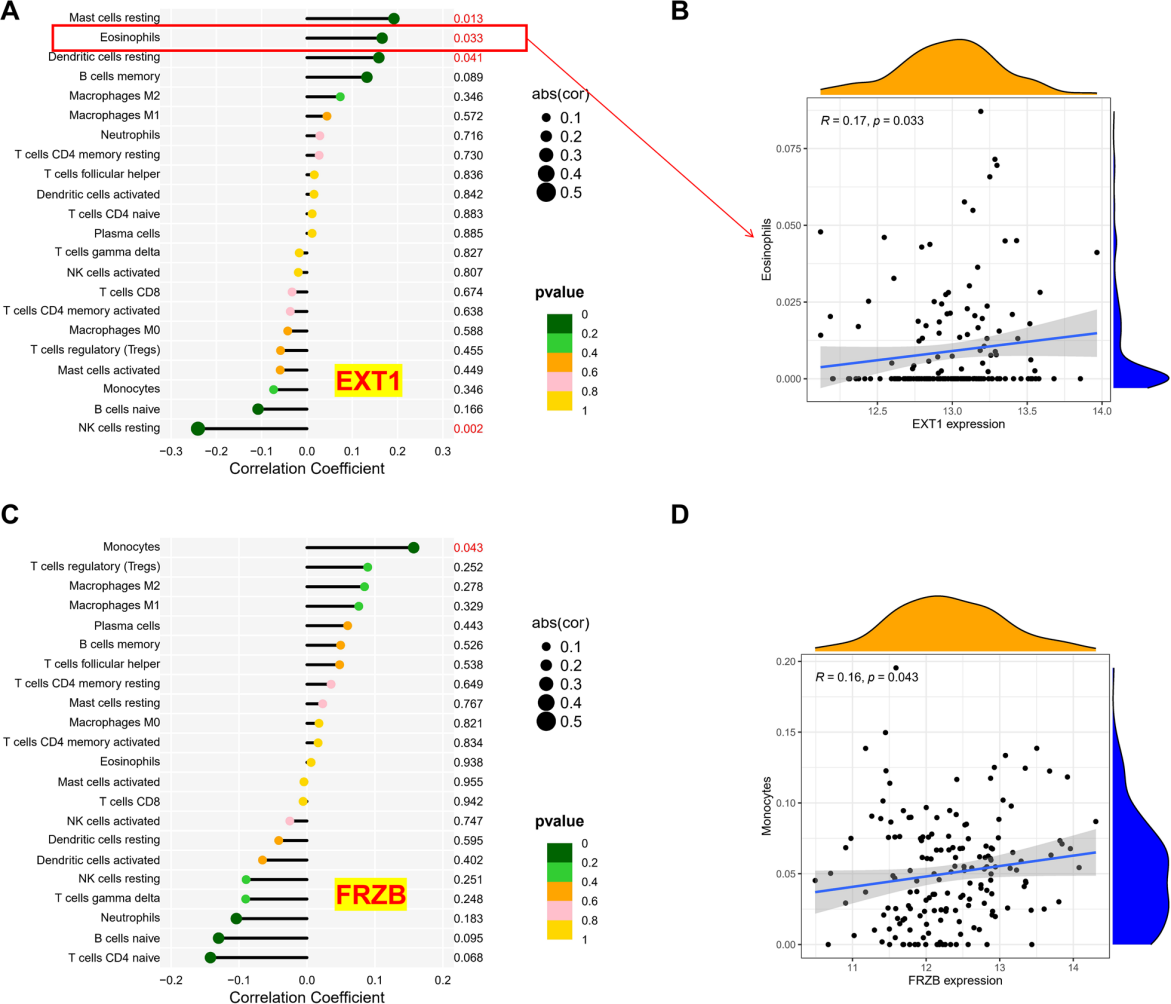
Correlation analysis between 22 kinds of immune cells and two hub genes. **A**, **B** EXT1; **C**, **D** FRZB

In DCM tissues, correlation analysis between 22 kinds of immune cells and 2 hub biomarkers was performed. Among them, EXT1 was negatively correlated with resting NK cells and positively correlated with resting dendritic cells, resting mast cells, and eosinophils (Fig. 7A). Figure 7B specifically shows the scatter plot of the correlation between EXT1 and core immune eosinophils. In addition, FRZB was positively correlated with monocytes (Fig. 7C, D).

### Validation of the expression levels of hub genes and immune cells in DCM

**Fig. 8:**
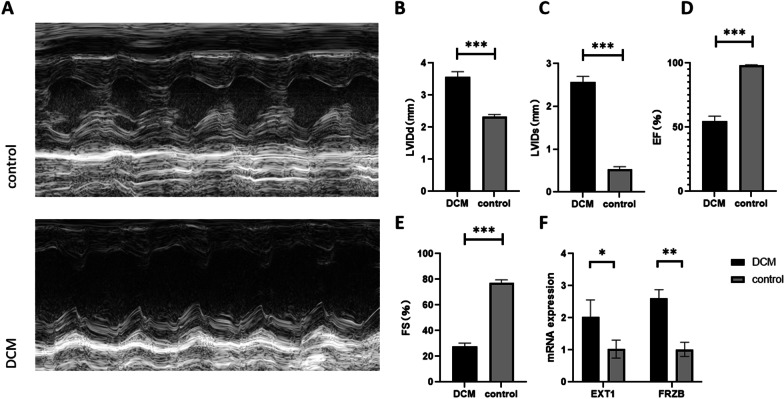
Representative echocardiographic images (**A**) and related cardiac function index (**B**-**E**) of the two groups. Validation of the expression levels of EXT1 and FRZB (**F**) in myocardial tissues of the DCM and control groups

**Fig. 9:**
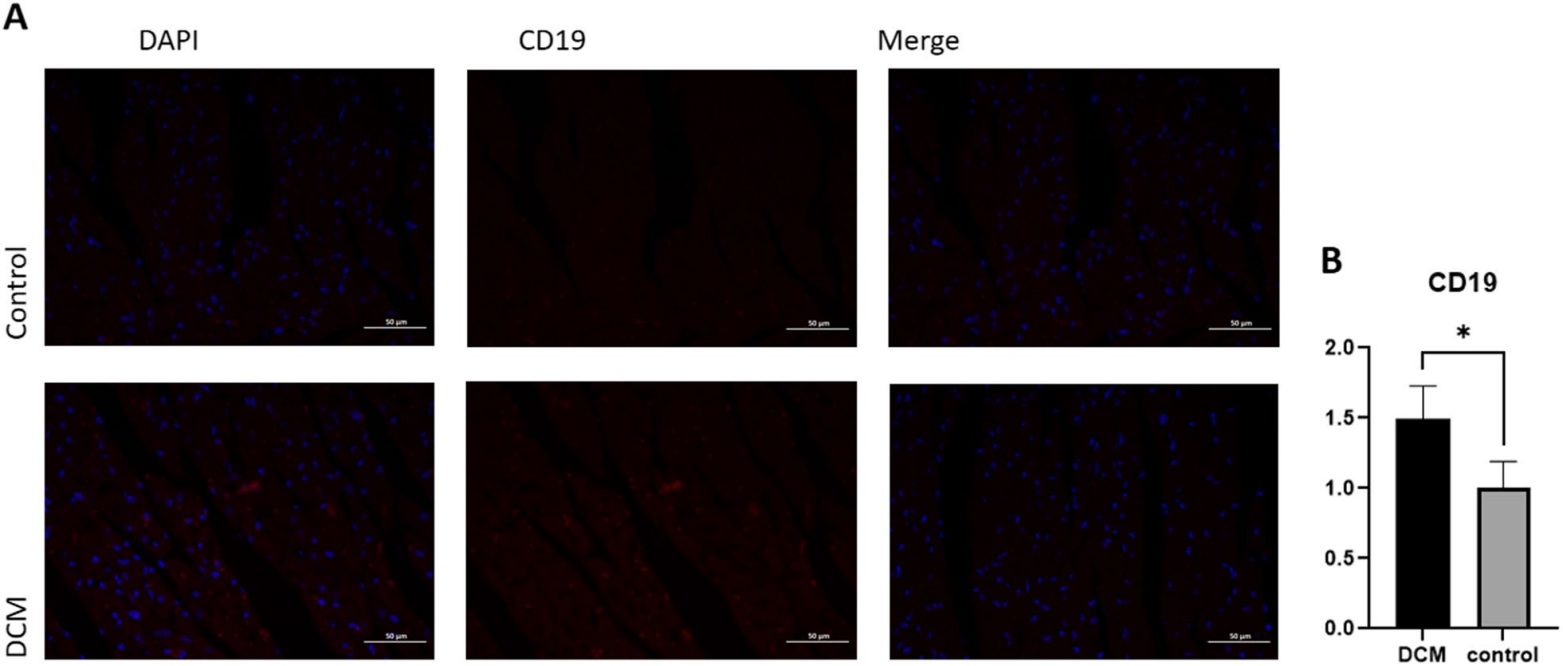
Representative immunofluorescence images (**A**) and IOD of CD19 (**B**) of the two groups

Animal experiments were performed to validate the expression of two hub genes and immune cells in myocardial tissue of healthy mice and DCM mice. The echocardiographic images (Fig. 8A) and related cardiac function index (Fig. 8B, E) showed that DOX induced dilated cardiomyopathy. The RT–PCR results showed that the mRNA expression levels of EXT1 and FRZB in DCM myocardial tissues were significantly higher than those in normal myocardial tissues, which was consistent with the results of the bioinformatics analysis (Fig. 8F). CD19 was used as a marker of naive B cells. The images of immunofluorescence (Fig. 9) showed that naive B cells increased in myocardial tissue in the DCM group.

## Discussion

As one of the leading causes of heart failure (HF), DCM is the most frequent indication for cardiac transplantation. DCM is a final common response of the myocardium to a quantity of genetic and environmental insults rather than a single disease entity. Contemporary studies using genetic screening show that up to 40% of DCM cases are genetically determined [3]. More than 50 genes related to sarcomeric proteins (MYH7, ACTC1, TNNT2, MYH6, MYBPC3), the cytoskeleton (TTN, DES, DMD, FLNC, NEXN, LDB3), ion channels (RYR2, SCN5A), the nuclear envelope (LMNA, TMPO) and intercellular junctions have been implicated in DCM.

Except for genetic factors, an important cause of acquired primary cardiomyopathy is myocarditis, which can lead to inflammatory dilated cardiomyopathy (IDC), a subtype of primary acquired DCM. Idiopathic-inflammatory, viral or autoimmune-mediated cardiomyocyte destruction mediated via several types of immune cells plays an important role in this process [28].

To investigate potential biomarkers for better detection and therapy, we integrated the gene expression profiles of GSE141910, GSE3585, GSE42955 and GSE79962, which contained 194 DCM samples and 187 normal samples. A total of 1861 upregulated genes and 1882 downregulated genes were identified. WGCNA, DO, GO, and KEGG enrichment analysis and multiple machine learning approaches were performed to identify the hub genes and specific immune cells.

We identified two hub genes, FRZB and EXT1. High expression of the two genes was significantly associated with DCM. FRZB (frizzled-related protein) functions as a modulator of Wnt signalling through direct interaction with Wnts and has a role in regulating cell growth and differentiation in specific cell types. It has been reported that FRZB serves as a key molecule in abdominal aortic aneurysm progression [29] and can decrease the growth and invasiveness of fibrosarcoma cells [30]. FRZB is also a muscle biomarker of denervation atrophy in amyotrophic lateral sclerosis [31]. EXT1 is an endoplasmic reticulum-resident type II transmembrane glycosyltransferase involved in the chain elongation step of heparan sulfate biosynthesis. Diseases associated with EXT1 include hereditary multiple exostoses, non-small-cell lung carcinoma and chondrosarcoma [32–34].

Weighted gene coexpression network analysis (WGCNA) and differential expression analysis were performed to identify gene panels related to DCM. Meanwhile, the CIBERSORT algorithm was used to estimate the immune cells in DCM tissues. Multiple machine learning approaches were used to screen the hub genes and immune cells. Finally, the diagnostic value of the hub genes was assessed by receiver operating characteristic (ROC) analysis.

Dilated cardiomyopathy caused by doxorubicin-induced myocardial injury represents a type of dilated cardiomyopathy caused by medicine, and whether the same results exist in dilated cardiomyopathy caused by other factors remains to be explored.

## Conclusion

In our research, 3743 DEGs were identified in DCM. Multiple machine learning approaches were used to screen the hub genes and immune cells. Two hub genes (FRZB and EXT1) could be used as promising biomarkers, and eosinophils, M2 macrophages, resting memory CD4 T cells and naive B cells may also affect the occurrence of DCM. The increase in two hub genes and naive B cells was validated in animal experiments (Additional files 1, 2, 3).

## Supplementary Information

**Additional file 1. Figure S1**. PCA before normalization PCA after normalization Figure S2 The TF-mRNA-miRNA network and potential candidate compounds targeting of two hub genes Figure S3 The histogram of the overall landscape of immune cell distribution and the heatmap of detail the correlation of 22 types of immune cells.

**Additional file 2.** DEGs between normal and DCM samples.

**Additional file 3.** Hub WGCNA genes between normal and DCM samples

## Data Availability

The gene expression profiles of GSE141910, GSE3585, GSE42955 and GSE79962 were downloaded from Gene Expression Omnibus (https://www.ncbi.nlm.nih.gov/geo/).

## Abbreviations

DCM: Dilated cardiomyopathy
DEGs: Differentially expressed genes
LV: Left ventricular
PPI: Protein–protein interaction
qRT-PCR: Quantitative real-time polymerase chain reaction
GEO: Gene expression omnibus
GO: Gene ontology
KEGG: Kyoto encyclopedia of genes and genomes
WGCNA: Weighted gene coexpression network analysis
TOM: Topological overlap matrix
SVM-REF: Support vector machine–recursive feature elimination
IDC: Inflammatory dilated cardiomyopathy
ROC: Receiver operating characteristic
