# Supplementary material for: Exploration of dilated cardiomyopathy for biomarkers and immune microenvironment: evidence from RNA-seq
Source: BMC Cardiovasc Disord. 2022 Jul 18;22:320. doi: 10.1186/s12872-022-02759-7 (PMC9290235; doi:10.1186/s12872-022-02759-7)
Supplement: Supplementary file 1 — Additional file 1. Figure S1. PCA before normalization PCA after normalization Figure S2 The TF-mRNA-miRNA network and potential candidate compounds targeting of two hub genes Figure S3 The histogram of the overall landscape of immune cell distribution and the heatmap of detail the correlation of 22 types of immune cells. [file 12872_2022_2759_MOESM1_ESM.docx]

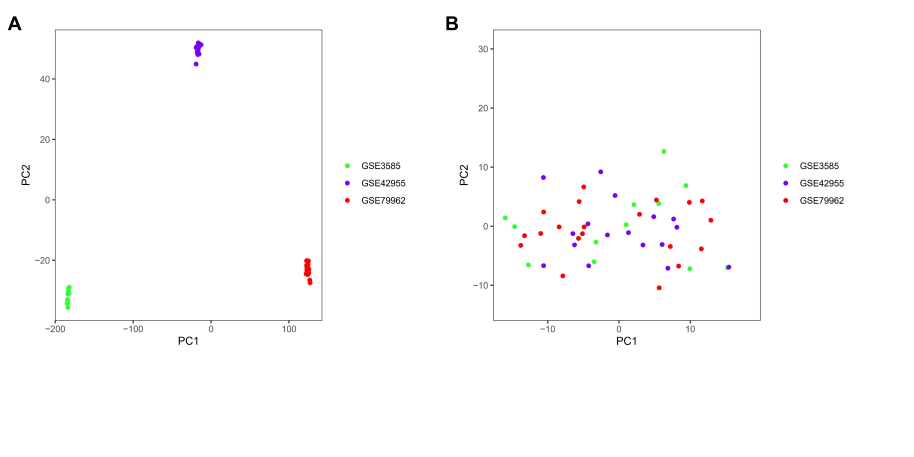


Figure S1 (**A**) PCA before normalization (**B**) PCA after normalization

**
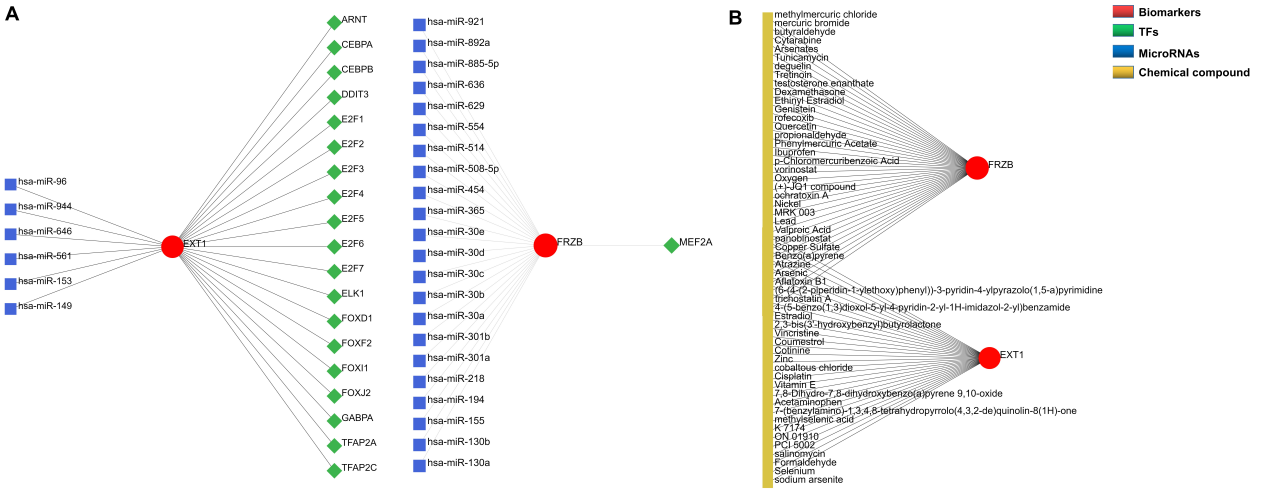
**

Figure S2Figure S2 The TF-mRNA-miRNA network(**A**) and potential candidate compounds targeting(**B**) of two hub genes


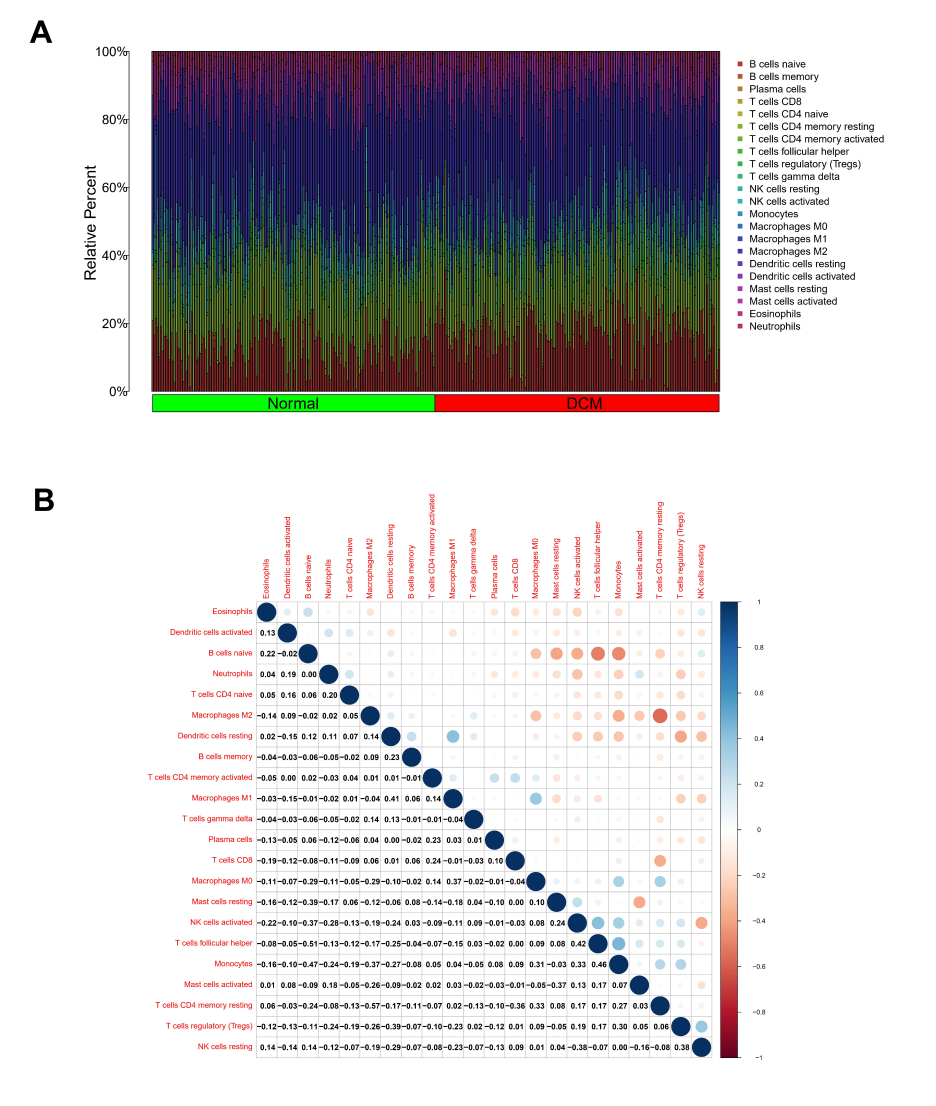


Figure S3 The histogram(**A**) of the overall landscape of immune cell distribution and the heatmap(**B**) of detail the correlation of 22 types of immune cells.
